# *Monanema joopi* n. sp. (Nematoda, Onchocercidae) from *Acomys (Acomys) spinosissimus* Peters, 1852 (Muridae) in South Africa, with comments on the filarial genus

**DOI:** 10.1051/parasite/2012194331

**Published:** 2012-11-15

**Authors:** K. Junker, K. Medger, H. Lutermann, O. Bain

**Affiliations:** 1 ARC-Onderstepoort Veterinary Institute Private Bag X05 Onderstepoort 0110 South Africa; 2 Department of Zoology and Entomology, University of Pretoria Private Bag X20 Hatfield 0028 South Africa; 3 Muséum National d’Histoire Naturelle, Parasitologie comparée, UMR 7205 CNRS CP52 61 rue Buffon 75231 Paris Cedex 05 France

**Keywords:** *Monanema joopi* n. sp., Onchocercidae, *Monanema*, parasitic nematodes, dermal microfilaria, *Acomys*, rodents, South Africa, *Monanema joopi* n. sp., Onchocercidae, *Monanema*, nématode parasite, microfilaire dermique, *Acomys*, rongeur, Afrique du Sud

## Abstract

*Monanema joopi* n. sp. is described from blood drawn from the heart of the murid *Acomys (Acomys) spinosissimus* in South Africa. It is characterised by a non-bulbous cephalic extremity, shared with only one of its five congeners, and a cylindrical tail with caudal alae and a spicular ratio of 2.7 in the male. As is typical for the genus, microfilariae are skin-dwelling. They are 185 to 215 micrometres long and have no refractory granules beneath their sheath. A key to the species of *Monanema* is presented and an amended generic description, based on the six currently known species, is proposed. Species of *Monanema* are primarily lymphatic and the low intensity of infection with *M. joopi* n. sp. in blood from the heart, might suggest that not all adults settle in the heart cavities. One might also consider that other, more susceptible rodents serve as hosts for this parasite as well. To date, the geographic range of *Monanema* includes North America, Africa and Australia, each with representatives of a different lineage. Given the present hypotheses on the evolutionary origin and subsequent migrations of rodents, we expect the origin of *Monanema* to be in the Palearctic-Oriental region.

## Abstract (fr)

Description de *Monanema joopi* n. sp., récolté dans le sang cardiaque d’*Acomys (Acomys) spinosissimus* en Afrique du Sud. L’espèce est caractérisée par une extrémité céphalique non bulbeuse, partagée avec un seul des cinq congénères, et chez le mâle, une queue cylindrique avec des ailes caudales et un rapport spiculaire de 2,7. Les microfilaires sont dermiques, ce qui est caractéristique du genre. Elles sont longues de 185-215 μm et n’ont pas de granules réfringents sous la gaine. Une clé des espèces de *Monanema* est présentée, ainsi qu’un amendement de la définition générique, basée sur les six espèces. Les espèces de *Monanema* sont fondamentalement lymphatiques et la faible infection par *M. joopi* n. sp. dans le sang cardiaque permet de suspecter d’autres localisations des adultes. Il se peut aussi que d’autres rongeurs plus réceptifs soient parasités. Actuellement, la distribution géographique de *Monanema* comprend l’Amérique du Nord, l’Afrique et l’Australie, et chaque région a des représentants de différentes lignées. D’après les hypothèses actuelles sur l’origine et les migrations ultérieures des rongeurs, nous pensons que *Monanema* est originaire de la région palearctico-orientale.

## Introduction

During an on-going ecological study investigating the ectoparasite and helminth assemblages of murids in South Africa, tiny filarial worms were collected from blood taken from the heart of the Spiny mouse *Acomys (Acomys) spinosissimus* (Peters, 1852). They represented a new species of *Monanema* Anteson, 1968 (Onchocercidae), a genus parasitic in rodents of which the microfilariae are not in the blood, but in the skin (Ko, 1972; Bianco & Muller, 1977; Bianco *et al.*, 1983; Bain *et al.*, 1985). In this paper, we give a morphological description of the new species and provide a synthetic analysis of the morphology and biology of all currently known species of *Monanema*. As a result, the definition of the genus was amended, and a hypothesis on its evolution proposed.

## Materials and Methods

*Acomys (A.) spinosissimus* was recovered from Goro Game Reserve (22° 58’ S, 29° 25’ E) in the Limpopo Province in South Africa. Filarial worms were fixed in 70 % ethanol. For morphological studies, worms were cleared in lactophenol and examined under a Wild compound light microscope equipped with a drawing tube. Caudal papillae are tentatively numbered following Chabaud & Petter (1961). Cross-sections were cut with a razor blade in order to study internal structures such as chords, muscles and oesophagus. Measurements were taken otherwise specified. The width of the buccal capsule was taken as its external diameter, measured at the base. Microfilariae were dissected from the uteri close to the vagina and cleared in lactophenol for further study. As earlier studies on *Monanema* indicated that the ear lobes are a predilection site for its skindwelling microfilariae (Ko, 1972; Muller & Nelson, 1975; El Bihari *et al.*, 1977; Bianco *et al.*, 1983; Bain *et al.*, 1985; Wanji *et al.*, 1990, 1994), ear snips were taken from the frozen carcasses of all infected animals and teased apart in lactophenol to check for microfilariae. Specimens have been deposited in the collection of the Muséum National d’Histoire Naturelle (MNHN), Paris, France (accession numbers 340 – 347 YU). Nomenclature of small mammals follows Wilson & Reeder (2005). Additional organs of some of the hosts that were infected with filariae could be examined at a later stage, using frozen carcasses. This was possible only in cases where these organs had not been dedicated to other studies. The frozen liver, lungs and wall of the caecum-colon of five, two and four animals, respectively, were examined, since adults had been reported from these sites in previous studies (Webster, 1967; Muller & Nelson, 1975; El Bihari *et al.*, 1977; Bain *et al.*, 1986; Wanji *et al.*, 1990).

## Results

One to two filarial worms were present in blood drawn from the heart of ten of 139 *A. (A.) spinosissimus*. The liver, lungs and wall of the caecum-colon of five, two and four of these ten hosts, respectively, did not contain any filariae. Microfilariae were found in ear snips of two of the hosts harbouring adult worms.

### *Monanema Joopi* n. sp. Junker & Bain (Figs 1, 2; Tables I-III)

**Fig. 1.**
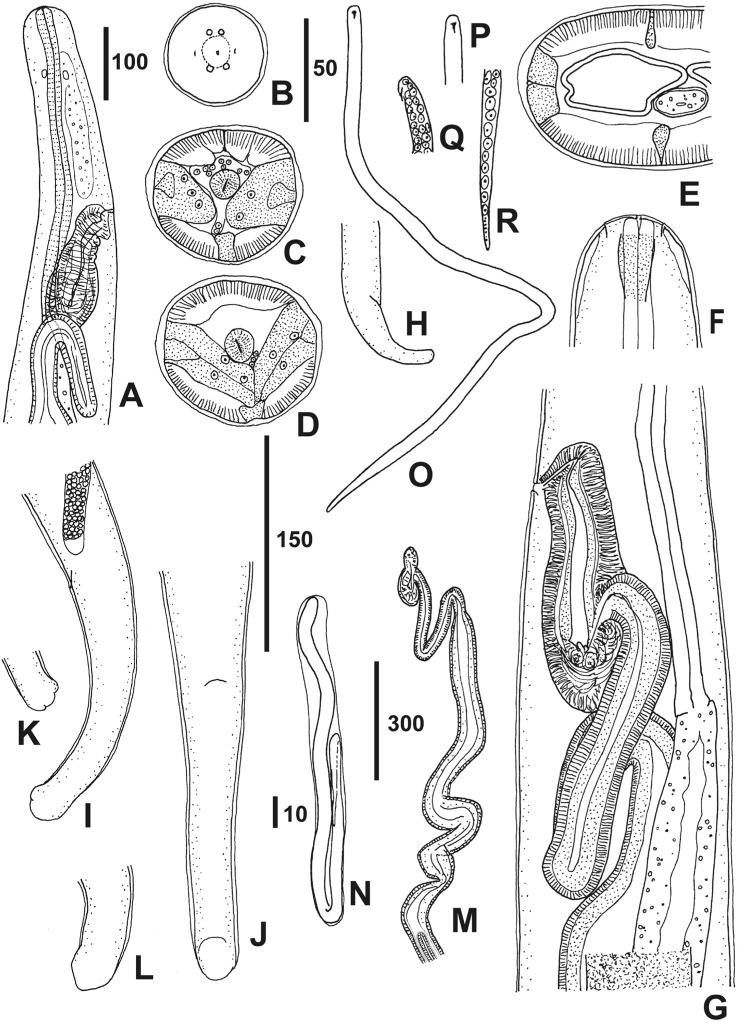
*Monanema joopi* n. sp., female. A, anterior region, right lateral view; B, head, in front view; C-E, three cross sections posterior to nerve ring, anterior to vulva, at midbody, respectively; F, anterior extremity, ventro-dorsal view; G, oesophageal-intestinal junction, vagina and anterior part of ovejector, left lateral view; H, tail, right lateral view; I & J, tails, left lateral view and ventral view, respectively; K & L, caudal extremities, right and left lateral views; M, ovejector and beginning of the two uteri, after dissection; N-R, microfilariae extracted from uteri; N, folded in sheath; O, exsheathed; P & Q, two anterior extremities with hook, dorso-ventral and left lateral view, respectively; R, last nuclei and granules at posterior extremity. Scales in μm: A, H, 100; B-F, I-L, 50; G, 150; M, 300; N-R 10.

Large parts of the specimens’ body were covered with patches of red blood cells. Slender worms, offwhite in colour. Body of both sexes tapering at ends but anterior extremity nearly cylindrical. Head not bulbous. Oesophagus not divided into muscular and glandular part, of nearly uniform diameter, posterior extremity slightly flattened at junction with intestine. Mouth opening tiny, round (Fig. 1B). Buccal capsule minute. Cuticle smooth.

**Fig. 2.**
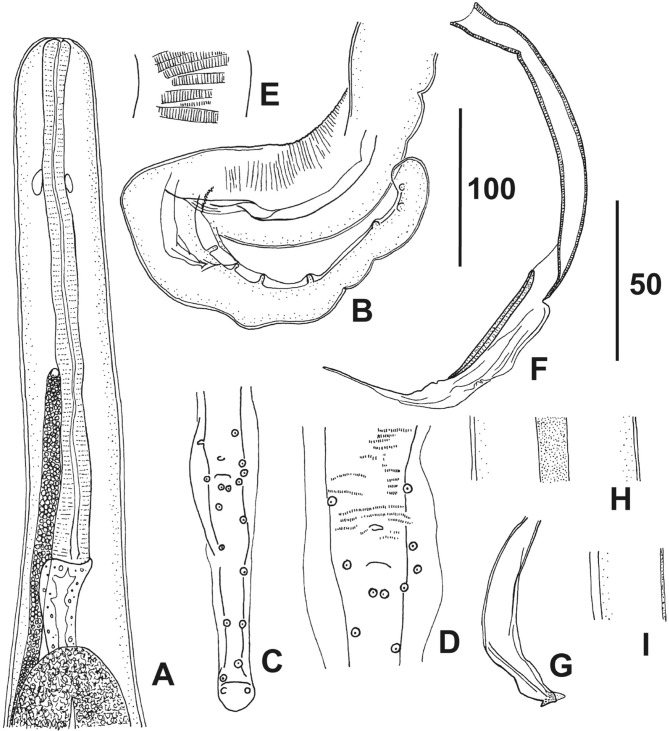
*Monanema joopi* n. sp., male. A, anterior region, dorso-ventral view; B, posterior region, ventral view anterior to cloaca but alae in lateral view; C, tail, ventral view; D, papillae and *area rugosa* near cloacal aperture, ventral view; E, *area rugosa*, 150 μm anterior to cloacal aperture, ventral view; F, left spicule, left lateral view; G, right spicule, right lateral view; H, lateral chord at mid-body, lateral view; I, cuticle thickened laterally, ventrodorsal view (half the width of worm drawn). Scales in μm: A-C, H, I, 100; D-G, 50.

Female (Fig. 1A-M; Tables I, III) posterior part narrower than anterior part. Cuticle slightly thickened laterally (Fig. 1C, D, E). Lateral chords thick; in lateral view, ventrally to the oesophagus, a peculiar cellular mass is observed (Fig. 1A), its origin is anterior to the nerve ring; in transverse section, this mass appears to be formed by the lateral chords which are directed ventrally (Fig. 1C), the left and right being joined in the median plane (Fig. 1D). One group of four head papillae observed, likely externolateral, arranged in dorsoventrally elongated rectangle; lateral amphids identified (Fig. 1B). Oesophagus with flattened lumen (not y-shaped; Fig. 1C, D), its posterior extremity at posterior level of vagina.

Vulva a longitudinal slit, at level of posterior half of oesophagus (Fig. 1A). Vagina: a short *vagina vera*, transverse, with flattened lumen; *vagina uterina* well-developed, directed posteriorly, with a chamber terminating in a sphincter, composed of epithelial cells (Fig. 1G). Ovijector: very thick, with a few loops, joining vagina near its mid-length. Opisthodelphic. Uteri running parallel. Tail: curved ventrally, especially tip; tip rounded and slightly bulbous, without appendages, often with irregular swellings, rarely lobulated (Fig. 1I-L). Phasmids not seen.

Microfilariae (Fig. 1N-R; Table III): uterine microfilariae folded once or twice, in loose-fitting sheath with obtuse ends; first fold usually in posterior half of body. No refractile granules in egg space between sheath and microfilaria. Body slender, posterior region tapering. Left cephalic hook, 2.5–3 long. No cephalic space, nuclei filling head anteriorly; nuclei terminating in single row at short distance from tip of tail. Terminal nuclei at times difficult to distinguish from fine granular material filling tip of tail. In skin, ten of 13 microfilariae were exsheathed. Dermal microfilariae (n = 4) were 190, 220, 225 and 228 long and 5, 5, 6 and 5 wide. The sheath (n = 2) was 132 and 147 long, respectively and 15 wide.

Male (Fig. 2A-I; Table II): posterior curled into four tight coils. Tail elongate, slender and cylindrical in ventral view, tip bulbous, without appendages or projections. Narrow caudal alae present, slightly more pronounced on level of cloaca. Caudal papillae: four pairs of pre- and paracloacal papillae (right papillae atrophied in pairs 2 and 4); pair 5 near ventral line; pairs 6–10 asymmetric, roughly evenly spaced on tail (Fig. 2C, D). *Area rugosa* on coiled part of posterior region, composed of narrowly spaced transverse bands of longitudinal crests (Fig. 2E), terminating approximately on level of cloacal aperture (Fig. 2D), not extending to tail. Spicules unequal and dissimilar. Left spicule with well cuticularized handle, followed by membranous lamina, ending in short filamentous tip. Right spicule short and robust, well cuticularized, with broad pointed tip and recurved hook. Gubernaculum absent.

**Table I.** Characteristics of female Monanema joopi n. sp. from *Acomys (Acomys) spinosissimus* Peters, 1852 in South Africa.

MNHN host/specimen number	340 YU/1	342 YU/1	343 YU/1	343 YU/2	344 YU/1	344 YU/2	344 YU/3	345 YU/1	346 YU/1	347 YU/1	347 YU/2
Body length (mm)	24.5	26.5	–	22.6	–	–	28.7	30.5	**29.7**	30.8	29.9
Maximum width	155	130	140	165	–	–	–	175	**150**	200	210
Buccal capsule length	–	–	–	1.7	–	–	2	2	–	2	–
Buccal capsule width	–	–	–	5	–	–	5	5	–	5.7	–
Oesophagus length	405	430	335		450	–	420	460	**492**	500	427
Apex to nerve ring	150	–	128	155	100	–	142		**110**	168	135
Apex to vulva	320	270	245	315	310	–	340	295	**370**	375	260
Length of vagina	–	–	125	–	–	–	185	185	–	–	165
Width of vagina	–	–	50	–	–	–	70	–	–	–	40
Tail length	150	160	–	150	–	180	175	145	**120**	155	120
Eggs	aborted	aborted	normal	normal	–	–	–	normal	**normal**	aborted	aborted
Microfilariae	few	few	**+++**	**+++**	–	–	–	**+++**	**+++**	none	none
Male gametes seen	none	none	none	**+++**	none	none	none	none	**none**	none	none

Holotype in bold characters. All measurements in micrometres, unless otherwise specified.

**Table II.** Morphological characteristics of the male of *Monanema joopi* n. sp. from *Acomys (Acomys) spinosissimus* Peters, 1852 in South Africa and its congeners.

	*Monanema* species					
	joopi n. sp.	marmotae	globulosa	nilotica	martini^b^	australe^b^
Authority and reference	341 YU/1; this paper	(Webster, 1967)	(Muller & Nelson,1975)	El Bihari, Hussein & Muller, 1977	Bain, Bartlett & Petit, 1986	Spratt, 2008
Host Family	Muridae	Sciuridae	Muridae	Muridae	Muridae	Muridae
Type host species	*Acomys (Acomys) spinosissimus*	*Marmota (Marmota) monax canadensis*	*Lemniscomys striatus*	*Arvicanthus niloticus*	*Arvicanthus niloticus*	*Melomys cervinipes*
Geographic origin	South Africa	Canada	Kenya	Sudan	Mali	Australia
Site of infection	heart	connective tissue of gall bladder and bile ducts	pulmonary arteries	heart, pulmonary arteries	lymphatic vessels of caecum-colon wall	lung parenchyma and terminal alveoli; hepatic blood vessels and lymphatics
No. of specimens examined	1	8	12	6–9	2	3

Body length (mm)	–	31–38	10.4–13.0	29–35	13.1; 13.2	24.3; 20.1; –
Maximum width	75	75–84	33–39	112–144	30; 20	49; 40; 44
Cephalic extremity	not bulbous	bulbous	bulbous	not bulbous	bulbous	bulbous
Oesophagus length	332	432–584	420–450	352–464	455; 480	600; –; –
Apex to nerve ring	92	approx. 60	120–150	123–130	120; 115	40; –; –
Left spicule (handle)	190 (105)	411–599 (156–181)	112–144 (–)	235–284	135 (65); 130 (65)	237 (97); 231 (94); 238 (97)
Right spicule	68	70–86	44–50	70–80	52; 45	49; 47; 52
Hook of right spicule	present	present	absent	present	double hook	present, keel-like
Spicular ratio (l/r)	2.7	5.5–6.7^c^	2.7	3.6	2.6; 2.9	4.8; 4.9; 4.6
Tail length	160	130–185	110–118	160^a^	130; 115	87; 84; 93
Shape of tip of tail	dilated	not dilated	not dilated	not dilated	not dilated	not dilated
Caudal alae	present	absent	absent	absent	present	present and particular
Position of area rugosa relative to cloaca	anterior	anterior and posterior	–	anterior	anterior and posterior	anterior

a measured on drawing;

b the first measurement given represents the holotype;

c calculation based on range of spicules. All measurements in micrometres, unless otherwise specified.

**Table III.** Morphological characteristics of the females and microfilariae of *Monanema joopi* n. sp. from *Acomys (Acomys) spinosissimus* Peters, 1852 and its congeners.

	*Monanema* species					
	joopi n. sp.	marmotae	globulosa	nilotica	martini^b^	australe^b^
Authority and reference	This paper	(Webster, 1967)	(Muller & Nelson,1975)	El Bihari, Hussein & Muller, 1977	Bain, Bartlett & Petit, 1986	Spratt, 2008
Female	n = 8–10	n = 10	n = 12	n = 15–23	n = 2	n = 3
Body length (mm)	24.5–30.8	67–92	7.6^b^–16	30–41	20.5; 22.5	33.2; –; –
Maximum width	130–210	108–139	33–47	160–224	45; 40	80; 74; 85
Cephalic extremity	not bulbous	bulbous	bulbous	not bulbous	bulbous	bulbous
Buccal capsule length	1.7	3.2	6	3	2.5; 2.5	2
Buccal capsule width	5.7	8.8^a^	9^a^	10^a^	5; 8	4.4^a^
Oesophagus length	335–500	404–760	380–470	448–544	460; 560	–
Apex to nerve ring	100–168	135–152	94	176–195	110; 110	–
Apex to vulva	245–375	336–544	130–330	304–392	390; 370	290; –; –
Tail length	120–180	–	130–140	208–272	85; 150	120; –; –
Appendages on tip of tail	no	no	yes	no	yes	no

Microfilaria	n = 15	–	–	n = 50	–	n = 1^d^
Site	uterus	abdominal fluid	skin of ears	skin of ears	uterus; dermal^e^	blood
Length	185–215	117–142	135–150	203–235	235–263; 250–288^e^	125
Maximum width	4–5	3.2–5.3	5.6–7.5	5–7	6; 6–8^e^	4
Length of oral hook	2.5–3	–	3^a^	2.5^a^	3.3^g^	2
Cephalic space	absent	3.8–5.5	5^a^	3–4	–	4.4^a^
Tail space	–	ca. 12	3^a^	7^a^	10^g^	–
Length of sheath	58–95 (n = 4)	108^a^	130^a^	167^a^	160^e^, ^f^	120^a^
Maximum width of sheath	8–12 (n = 4)	20^a^	15^a^	16^a^	12^e^, ^f^	15^a^
Fit of sheath	loose	loose	loose	loose	–	loose
Granules in sheath	absent	absent	10–11	17–34	11–26^f^	absent

a measured on drawing;

b immature specimen;

c the first measurement gien represents the holotype;

d from *Uromys (Uromys) caudima- culatus* (Krefft, 1867);

e the last set of measurements taken from Bain *et al.* (1985);

f observed on live specimens;

g a single specimen measured. Host details and geographic distribution listed in Table II. All measurements in micrometres, unless otherwise specified.

Type host: *Acomys (Acomys) spinosissimus* Peters, 1852 (Muridae).

Type locality: Goro Game Reserve (22° 58’ S, 29° 25’ E), Limpopo Province, South Africa. Collection date: 20.02.2008.

Site of infection: blood drawn from cardiac cavities. Prevalence and intensity: prevalence was 7.2 %. Five hosts harboured a single worm, five hosts yielded two worms each

Type material: 346 YU; holotype female. Deposited in the MNHN collection.

Additional material: Collected from January to August 2008. All specimens deposited in the MNHN collection. 340 YU; entire female. 342 YU; entire female. 343 YU; two entire females, both burst posteriorly. 344 YU; entire female, anterior fragment (used for apical view) and posterior fragment of female. 345YU; entire female. 347YU; two entire females. 341YU; entire male, broken into anterior and posterior part during preparation of drawing.

Etymology: the new species is named after Prof. Joop Boomker in recognition of his vast contribution to our knowledge of the helminth fauna of South African wildlife.

### Taxonomic Discussion

The filariae described herein possess the long tail as well as the unequal and dissimilar spicules typical for Onchocercinae. Based on adult characters and skin-dwelling microfilariae in a loose-fitting sheath, they were assigned to *Monanema* (Chabaud & Bain, 1976; Anderson & Bain, 1976; Spratt, 2008). Presently, the genus comprises five species, described from the following type hosts. *Monanema marmotae* (Webster, 1967) Anteson, 1968 (= *Ackertia marmotae* Webster, 1967) was described from the sciurid *Marmota (Mar- mota) monax canadensis* (Erxleben, 1777) [= *Marmota monax canadensis* (Erxleben, 1777)] in Canada and the remaining four from murid hosts. *Monanema globulosa* (Muller & Nelson, 1975) was reported from *Lemniscomys striatus* (Linnaeus, 1758) in Kenya, *Monanema nilotica* El Bihari, Hussein & Muller, 1977 from *Arvicanthis niloticus* (Geoffroy, 1803) [= *A. niloticus testicularis* (Sundevall, 1843)] in Sudan, *Monanema martini* Bain, Bartlett & Petit, 1986 from *A. niloticus* in Mali, and *Monanema australe* Spratt 2008, from *Melomys cervinipes* (Gould, 1852) in Australia (Tables II, III; Webster, 1967; Muller & Nelson, 1975; El Bihari *et al.* 1977; Bain *et al.,* 1986; Spratt, 2008). All these are distinct from the current specimens in a number of characters, listed below and in Tables II & III. In addition, the hypertrophy of the lateral chords, joining ventrally in the anterior region, as seen in the current specimens (Fig. 1C, D), has not been described for any of the above species.

*Monanema marmotae*: head bulbous; external labial and cephalic papillae arranged in two squares; females more than twice as long (67–92 mm *vs* 24.5–30.8 mm); male tail conical in ventral view, without caudal alae; *area rugosa* extending to tail; left spicule approximately two to three times longer (411–599 *vs* 190), spicular ratio about twice as high (5.5–6.7 *vs* 2.7); microfilariae shorter (117–142 *vs* 185–215) (Webster, 1967).

*Monanema australe:* head bulbous; tip of female tail not bulbous; male tail twice shorter, conical in ventral view, with prominent paracloacal alae ornamented with small rugosities at base and with three pairs of adcloacal, pedunculate, laterally directed papillae; left spicule longer (231–238 *vs* 190), right spicule shorter (47–52 *vs* 68), resulting in higher spicular ratio (4.6–4.9 *vs* 2.7); microfilariae shorter (125) (Spratt, 2008).

*Monanema globulosa:* head bulbous [we noted discrepancies between the scale bar, illustration and text concerning Fig. 1 in Muller & Nelson (1975)]; females shorter (7.6–16 mm *vs* 24.5–30.8 mm), with two pairs of small appendages on tail; male tail conical in ventral view, without caudal alae; caudal papillae more symmetrically arranged, with three regular pairs of precloacal papillae; left and right spicules shorter (112–144 and 44–50 *vs* 190 and 68, respectively), right spicule without hook; microfilariae shorter (135–150 *vs* 185–215), 10–11 refractory granules beneath sheath (Muller & Nelson, 1975).

*Monanema martini:* head bulbous; females shorter (20.5–22.5 mm *vs* 24.5–30.8 mm), tip of tail with several conical projections; caudal papillae grossly symmetrical; *area rugosa* extending to mid-tail; both left and right spicule shorter (135 and 130, and 52 and 45 *vs* 190 and 68, respectively), right spicule with double hook; microfilariae larger (235–263 *vs* 185–215), 11–26 refractory granules beneath sheath (Bain *et al.,* 1986).

*Monanema nilotica:* external labial and cephalic papillae arranged in two squares; female tail longer (208–272 *vs* 120–180); male tail conical in ventral view, without caudal alae; all caudal papillae paired and symmetrically arranged; two precloacal pairs; spicular ratio higher (3.6 *vs* 2.7); microfilariae larger (203–235 *vs* 185–215), 13–34 refractory granules beneath sheath (El Bihari *et al.,* 1977).

### Identification Key to the Species of *Monanema* Anteson, 1968

1-(4) Cephalic extremity not bulbous.

2-(3) Male tail conical in ventral view, caudal alae absent. Spicular ratio 3.6. 17–34 refractory granules beneath sheath of microfilariae. Microfilariae 203–235 long.

*M. nilotica* El Bihari, Hussein & Muller, 1977

3-(2) Male tail cylindrical in ventral view, caudal alae present. Spicular ratio 2.7. No refractory granules beneath sheath of microfilariae. Microfilariae 185–215 long.

M. joopi n. sp.

4-(1) Cephalic extremity bulbous.

5-(8) Female tail with appendages or projections. Spicular ratio 2.6–2.9. Filamentous part of lamina of left spicule not longer than membranous part. Refractory granules beneath sheath of microfilariae.

6-(7) Female tail with several conical projections. Male tail cylindrical, narrow caudal alae present. Right spicule with double hook. 11–26 refractory granules beneath sheath of microfilariae. Microfilariae 235–288 long.

*M. martini* Bain, Bartlett & Petit, 1986

7-(6) Female tail with two pairs of small appendages. Male tail conical, caudal alae absent. Right spicule without hook. 10–11 refractory granules beneath sheath of microfilariae. Microfilariae 135–150 long.

*M. globulosa* Muller & Nelson, 1975

8-(5) Female tail without appendages or projections. Spicular ratio > 4.6.Filamentous part of lamina of left spicule longer than membranous part. No refractory granules beneath sheath of microfilariae.

9-(10) Male tail conical in ventral view, caudal alaeabsent. *Area rugosa* anterior and posterior tocloaca. Spicular ratio 5.5–6.7. Membranous part of lamina of left spicule reduced. Right spicule with simple hook. Microfilariae 117–142 long.

*M. marmotae* Webster, 1967

10-(9) Male tail conical in ventral view, paracloacalalae prominent with three pairs of laterallydirected adcloacal pedunculate papillae. *Area rugosa* anterior to cloaca, but cuticular rugosities at base of paracloacal alae. Spicular ratio 4.6–4.9. Right spicule with pair of keel-like structures. Microfilariae 125 long.

*M. australe* Spratt, 2008

## Discussion

**Fig. 3.**
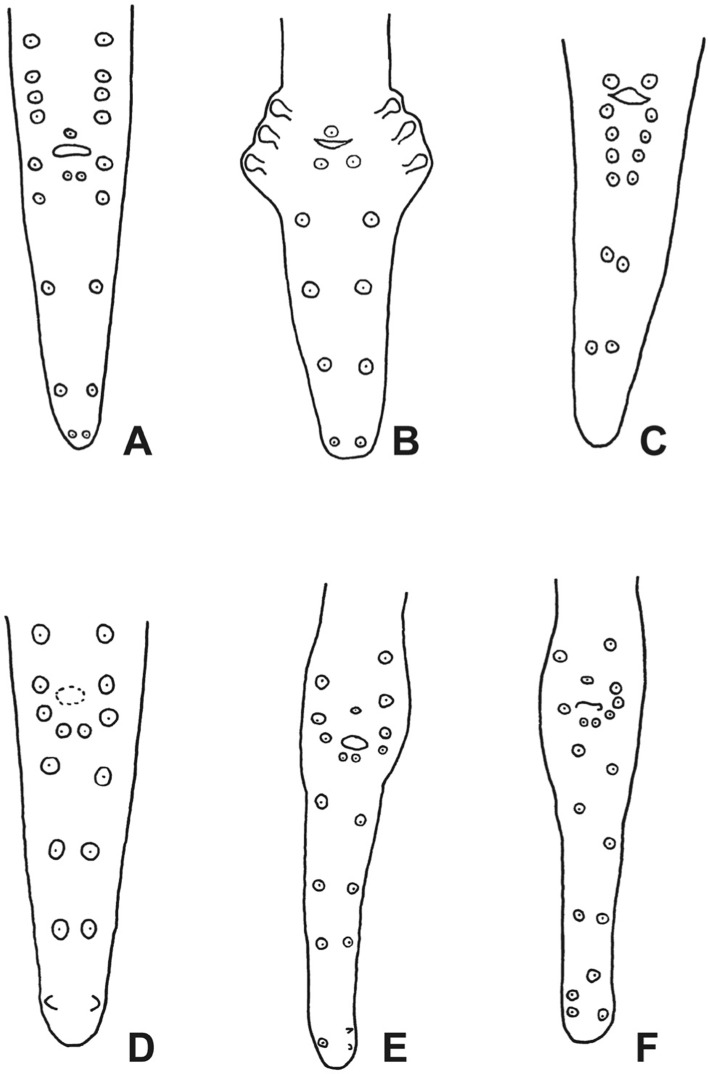
Schematic representation of the arrangement of the caudal papillae of *Monanema* species. A, *Monanema marmotae* (reconstructed from lateral view illustrated in Webster, 1967); B, *Monanema australe* (redrawn after Spratt, 2008); C, *Monanema nilotica* (redrawn after El Bihari *et al*., 1977); D, *Monanema globulosa* (redrawn after Muller & Nelson, 1975); E, *Monanema martini* (redrawn after Bain *et al*., 1986); F, *Monanema joopi* n. sp.

*Monanema* was created by Anteson (1968, unpublished thesis; in Chabaud & Bain, 1976) for the species then known as *Ackertia marmotae* Webster, 1967 because, contrary to *Ackertia* Vaz, 1934, its male has several pairs of postcloacal papillae. The genus *Monanema* was accepted by Anderson & Bain (1976), Chabaud & Bain (1976) and Bain *et al.* (1982). In the characters of *Monanema* listed by these authors, the undivided oesophagus and caudal papillae on the tail were consistent, but other characters (buccal capsule, spicular ratio, head shape) changed, to accommodate the increasing number of species described. Taking into account the characteristics of the six presently described species, we propose the following amended generic definition for *Mona- nema:* buccal capsule small (1.7–6 long, 4.4–10 wide); oesophagus not divided into muscular and glandular part; vulva on level of posterior half of oesophagus; vagina large with chamber and sphincter; absence of caudal appendages on tip of male tail; 7–10 pairs of caudal papillae (Fig. 3), disposed anterior to cloaca and, grossly equidistant, along length of tail; first pair of postcloacal papillae close to midventral line; spicular ratio 2.7–6.7; microfilariae folded in loosefitting sheath with obtuse extremities; microfilariae skin-dwelling.

The life cycles of three of six species have been elucidated. The intermediate hosts are ixodid ticks for *M. marmotae* (Ko, 1972), *M. globulosa* (Bianco & Muller, 1983) and *M. martini* (Bain *et al.* 1985; Petit *et al.,* 1988). It is thus expected that ixodids will also transmit *M. joopi* n. sp. and the remaining two species. Two ixodid ticks were collected from *A. (A.) spinosissimus* examined during the current study, *Rhipicephalus simus* Koch, 1844 and *Rhipicephalus follis* Donitz, 1910. While they are likely vectors, their role in the life cycle of *M. joopi* n. sp. remains to be confirmed.

Sites of infection for the species of *Monanema* are diverse, including the lungs, heart cavities, lymphatics of liver and caecum-colon. For a given species, as for example *M. martini,* filariae can settle in different places, the lymphatics and, less commonly, in the pulmonary arteries (Wanji *et al.,* 1990; Vuong *et al.,* 1991). In fact, it seems that species of *Monanema* are primarily lymphatic as suggested by the works of Wanji *et al.* (1990) who also demonstrated that lymphatic infective larvae and adults could be passively drawn back to cardio-pulmonary sites by the lymph flow when altered (Bain & Babayan, 2003). The low intensity of infection seen in *M. joopi* n. sp., the high number of aborted eggs in females and the fact that on no occasion males and females were recovered together, suggests that not all adults settle in the heart cavities. However, no adults were recovered from any of the other sites examined (liver, lungs, caecum- colon). This also suggests that other, more permissive murids might contribute to the maintenance of *M. joopi* n. sp. in nature.

*Monanema* has few representatives but a wide geographic distribution (Tables II, III; Spratt, 2008). The host range of *Monanema* is restricted to rodents, with one species in a Nearctic sciurid and five species in murids, of which four in Africa and one in Australia. The trends of morphological evolution in the *Dipetalonema* line (Chabaud & Bain, 1976), indicate that a bulbous head, head papillae that are arranged in a dorsoven- trally elongated rectangle [in *M. martini* as early as in the infective stage (Bain & Chabaud, 1986)], a high spicular ratio, pairs of caudal papillae that are asymmetrically arranged and a reduced number of caudal papillae are evolved characters. We thus propose the following hypothesis for the relationships among the species of *Monanema.* The single parasite in a sciurid, *M. marmotae,* represents a line with a combination of primitive and derived characters (Webster, 1967). Among the species parasitic in murids, the four species in Africa form another line, sharing the primitive character of a small spicular ratio (≤ 3.6), but diversified with respect to other characters: in West and West-Central Africa, *M. martini* (Bain *et al.,* 1985; unpublished data); in South Africa, *M. joopi* n. sp.; in East Africa, *M. nilotica* (El Bihari *et al.,* 1977); and *M. globulosa* from mountains in Kenya (1,500 meters of altitude). In Australia, *M. australe* represents a third evolutionary lineage (Spratt, 2008).

The Afrotropical region has the highest number of species but is unlikely the place of origin of *Monanema.* Rodents most likely originated from Eurasia (Wilson & Reeder, 2005; Jansa *et al.,* 2009). During the Miocene/Pliocene, murids dispersed into Africa via the Arabian Peninsula (Winkler, 1994) and into Australia, via South-East Asia and New Guinea (Godthelp, 2001). Marmots, on the other hand, first arose in North America and spread into Eurasia via the Bering land bridge (Steppan *et al.,* 1999). The origin of *Monanema* is probably the Palaearctic-Oriental region. If a protocol for the detection of dermal microfilariae was included in helminth diversity studies, more species of *Mona- nema* would likely be discovered. Particularly, the important finding by Spratt (2008) of a species in an Australian murid, Melomys Thomas, 1922, reveals that representatives of *Monanema* can be expected in the Indomalayan and Australasian region. To date, only the 12s rDNA sequence of *M. martini* is known (Ferri *et al.,* 2011). In future, additional molecular studies might help to elucidate the phylogenetic relationships within this genus and to establish its taxonomic position within the Filarioidea.

Filariae with dermal microfilariae are rarely detected. Even in animals as well-studied as dogs and rodents, several new species have been reported in the past ten years, for example *Onchocerca lupi* (Sréter & Szell, 2008) and *Cercopithifilaria* spp. (Otranto *et al.*, 2011). This is examplified in *Monanema* in which only six species have been described in over 40 years, although their current geographical and host distribution suggests a much higher diversity.
